# Surgical treatment of liver metastases of gastric cancer: state of the art

**DOI:** 10.1186/1477-7819-10-157

**Published:** 2012-08-03

**Authors:** Fabrizio Romano, Mattia Garancini, Fabio Uggeri, Luca Degrate, Luca Nespoli, Luca Gianotti, Angelo Nespoli, Franco Uggeri

**Affiliations:** 1 Department of Surgery, San Gerardo Hospital- University of Milano Bicocca, Via Donizetti 106, Monza 20052, Italy

**Keywords:** Gastric cancer, Liver resection, Metastases, Prognosis

## Abstract

**Background:**

The prognosis of patients with liver metastases from gastric cancer (LMGC) is dismal, and little is known about prognostic factors in these patients; so justification for surgical resection is still controversial. Furthermore the results of chemotherapy for these patients are disappointing. The purpose of this study was to review recent outcomes of hepatectomy for LMGC and to determine the suitable candidates for surgery, assessing the surgical results and clinicopathologic features. Moreover we compare these results with those obtained with alternative treatments.

## Introduction

Gastric cancer is the fourth most common cancer worldwide and account for 1.5% of all new diagnoses and 5.2% of all cancer deaths [1,2]. At the time of diagnosis 35% of patients present with evidence of distant metastases and 4% to 14% have metastatic disease to the liver [3,4]. Although the effectiveness of liver resection for metastatic colorectal cancer has been already established [5-7], reports of hepatic resection for liver metastases of gastric cancer are rare and its significance is still controversial [8]. In fact a number of studies reported that the effect and benefit of hepatic resection for either synchronous or metachronous gastric hepatic metastases (LMGC) on survival was dubious [9]. Furthermore the surgical indications for liver metastases of colorectal cancer have been expanded to include all technically resectable metastases numbering 4 or more [10]. On the contrary, the surgical indications for liver metastases of gastric cancer must be carefully determined because of the more severe biologic nature of this disease [11].

Most patients with gastric cancer with concomitant liver metastases are excluded from candidates for curative surgery accompanied with hepatic resection due to incurable simultaneous factor such as peritoneal dissemination, widespread lymph nodal metastases, and direct invasion to adjacent structures [12]. In fact LMGC often represent only a part of a generalized spread of the primary tumor (‘the tip of the iceberg’). Furthermore very few patient with LMGC are good candidates for liver surgery due to multiple, scattered, bilobar lesions [13]. Patients with isolated metastases are unusual, accounting for 0.5% of cases in the Linhares’s series [14]. On the other hand metastatic liver involvement, which occurs in up to 50% of patients with gastric cancer, makes long-term survival without treatment impossible, with a median survival of 6 months. These data show growth to 7 to 15 months with chemotherapy schedules. There are no adequate large prospective studies detailing the natural history of metastatic gastric carcinoma and long-term survival. However, two small randomized trials compared best supportive care *vs.* combination chemotherapy and found that no patients treated with supportive care lived for >1 year [15,16]. Survival data for patients with metastatic gastric cancer (MGC) to the liver only are also limited. In a study analyzing 643 patients enrolled in five separate chemotherapy trials by the Japanese Clinical Oncology Group (JCOG), 5-year survival for patients with metastases confined to the liver and treated with systemic therapy alone was 1.7% [17]. Palliative chemotherapy using various regimens has been widely used as the treatment of choice, but only modest improvements in overall survival have been observed, with median survival increasing from approximately 3 months to 7 to 15 months. Long-term survival is rarely reported [18-20]. In particular, considering the few trials evaluating systemic chemotherapy in the subset of patients with liver-only metastatic involvement, 5-year survival rates do not reach 2% [21].

Baba *et al*. have shown that the outcome for patients with non-curative resection for advanced gastric cancer is extremely poor [22], while several authors have reported on their limited experiences of surgical complete resection of the metastatic tumors in selected patients of LMGC, with 5-year survival rates ranging from 0% to 38% [23-25], considering patients with liver metastases as sole metastatic site. However, considering survival performances extrapolated from a cohort of 1,452 patients submitted to hepatic resection for non-colorectal non-endocrine liver metastases, Adam *et al*. [26] observed that metastases from gastric adenocarcinoma performed in an intermediate way, ranking 10th in a list of 18 primaries. Moreover a recent review of the literature about LMGC report a median 1-, 3-, and 5-year survival on 436 patients of 62%, 30%, and 26.5%, respectively, and a median survival of 17 months [27]. So even if the percentage of patients who may benefit from resection is probably small, only surgery is able to obtain long-term survival. Therefore the determination of selection criteria for hepatic resection and conditions for long-term survival after hepatectomy for LMGC are crucial.

We reviewed the literature in which numerous factors were examined and their relationship to outcome assessed, in order to determine the benefits and the limits of hepatic resection for gastric metastases and to identify selection criteria for good outcome. In fact identification of prognostic factors that predict outcome following surgical resection of gastric hepatic metastases should assist in identification of patients most likely to benefit from this intervention, or more importantly, assist in identification of patients unlikely to benefit. Moreover, we analyze a more recent study which compares the surgical approach to other treatment modalities such chemotherapy or radiofrequency.

## Indications

A literature review of papers written in English was performed. The criteria for hepatic resection offered by Okano *et al.*[28] are broadly defined: hepatic resection is indicated in patients: (1) with synchronous metastases who have no peritoneal dissemination or other distant metastases; and (2) with metachronous metastases, but no other recurrent lesion. Ambiru *et al.*[9] added a third criterion, that is, complete resection of hepatic metastases with acceptable postoperative hepatic function. In a recent report by Roh *et al.*[29], hepatic resection is said to be indicated only in patients with hepatic metastases in one lobe of the liver without peritoneal dissemination, hilar node metastases, or distant metastases. Criteria actually accepted for resection of hepatic metastases from gastric cancer are now as follows: (1) good control of the primary tumor and complete resection of primary tumor and lymph nodes involvement in synchronous disease; (2) no signs at preoperative work-up of disseminated diseases, hilar lymph nodes metastases, peritoneal dissemination, or extrahepatic metastases; (3) complete resection of hepatic metastases (macroscopically no residual tumor). Following these selection criteria Ochiai *et al*. [30] found a hepatic resection incidence of 21 in 6,540 patients (0.3%) with a gastric cancer who underwent a gastrectomy. Saiura *et al.*[31] found an incidence of 10 in 1,807 similar patients (0.6%), and Okano *et al.*[27] found an incidence of 19 in 807 patients (2.4%). A recent literature review reported only 229 liver resections for LMGC, maybe reflecting an *a priori* passive attitude toward these patients. Some studies report a classification of degree of liver metastases in patients with LMGC according to the Japanese Classification of Gastric Carcinoma (Table1) [32]. Studies in which synchronous *en bloc* resections of gastric cancer directly invading the liver were performed were not included in this review. In each study regarding patients with metachronous liver metastases the resection of the primary gastric cancer was categorized as curative or the studies were excluded from our analysis. Curative resection was defined by removal of all macroscopically detectable disease and microscopically clear resection margins (R0). Synchronous liver metastases were defined by detection before or during surgery in each study, or within 3 months after primary tumor resection. The following clinicopathologic factors were analyzed in the literature revision to underline their influence on patient’s outcome: age; gender; status of serosal invasion; histologic differentiation of the primary tumor; status of lymph nodes metastases; temporal relationship of metastases with primary disease (synchronous or metachronous); tumor distribution; size and number of liver metastases; classification of hepatic metastases according the Japanese Gastric Cancer Association proposal (Table1), type of hepatic resection; surgical margin and completeness of the resection; presence of pseudocapsule between metastases and liver parenchyma, histologic differentiation, and vascular invasion. These factors were then divided into three main categories.

1. Predictive of outcome related to primary tumor

2. Predictive of outcome related to metastases

3. Predictive of outcome related to surgery

**Table 1:** Classification of hepatic metastases from gastric cancer as proposed by the Japanese Gastric Cancer Association, 1998

**H-O**	**No liver metastases**
H-1	Liver metastases limited to one lobe of the liver
H-2	Isolated diverse metastases in both lobes of the liver
H-3	Multiple distributed metastases in both lobes of the liver

Liver surgical procedures were classified as anatomic resection (segmentectomy and lobectomy) or limited resection (all resections less extensive than segmentectomy). Moreover we analyzed literature regarding prognostic factors according to two main categories of papers: papers with selected or unselected populations.

### Prognostic factors

In the review of the literature hepatectomy was indicated in only 0.4% to 1% of gastric cancer patients with liver metastases. Unfortunately, most hepatic metastases from gastric adenocarcinoma are multiple, bilateral, and combined with peritoneal or lymph nodes metastases, which directly invade adjacent organs precluding a radical surgical approach. At specialized treatment centers, the proportion of surgery for hepatic metastases of gastric cancer is 7% to 12% of hepatic resection for all types of hepatic malignancies [33]. The resectability rate is low and is approximately 10% of cases, and it seems to be the same in cases of metachronous or synchronous metastases [34].

The effectiveness of hepatic resection has not been well-defined. In addition, the clinicopathologic characteristics related to the prognosis of gastric cancer with hepatic metastases have not been comprehensively identified. Nevertheless the presence of hepatic metastases is a statistically significant poor prognostic factor for patients with gastric cancer [35]. The cumulative survival rate reported in early studies was generally poor, reflecting a generalized disease. Elias *et al*. showed that the 3-year survival after hepatic resection was less than 20% [36]. In recent series the 1-year survival rate ranged from 42% to 90% and 5-year survival rate from 0% to 38% (Table2). The long-term results after liver resection for metastases from gastric cancer show a wide range (Table2). Most studies concerning this issue come from Japan and the reported long-term survival rates exceed 30% in some series [24,27]. In contrast, in the western study from Zacherl *et al.* none of the patients survived 5 years after resection [32].

**Table 2:** Literature analysis regarding hepatectomy for liver metastases from gastric cancer

**Author year**	***n***	**Period**	**Resection criteria**	**Resectability rate %**	**S/M**	**TG/STG**	**Major/Minor liver surgery**	**Solitary**	**Multiple Uni/Bilob**	**R1%**	**Overall survival % 1, 3, 5 years**	**Long-term survivors**	**Recurrence**	**Follow-up (months)**
1994 [30]	21		No extrahepatic No carcinosis R0	Na	13/8	Na		14	7	0	19	19% (4)		
1997 [43]	21		No extrahepatic No carcinosis R0	Na	11/10		5/16	7	14 11/3	Na	45 19 11	24% (5)	76.1%	n
2001 [23]														
	17	1990-1997	No extrahepatic											
No carcinosis														
R0	Na	7/10	na	6/11	8	9 4/5	18%	47 22 0	0	76%	Na			
2001 [9]														
	40	1975-1999	No extrahepatic											
No carcinosis														
R0	Na	18/22	19/21	21/19	19	21								
5/16	0	70 28 18	15% (6)	75%	88									
2001 [45]														
	10	1979-1999	Na	Na	3/7	3/7	6/4	6	4					
2/2	Na	60 20 20	10% (1)	80%	10-240									
2002 [33]														
	15	1980-1999	No extrahepatic											
No carcinosis														
R0	Na	10/5	9/6	3/12	8	7								
2/5	33	35.7 14.3 0	0	90%	Na									
2002 [31]														
	10	1981-1998	No extrahepatic											
≤ 3 segments	15.6%	7/3	Na	6/4	5	5								
4/1	40%	65 38 20	20% (2)	80%	1-68									
2002 [28]														
	19	1986-1999	No extrahepatic											
No carcinosis														
R0	17%	13/6	na	7/12	10	9								
2/7	0	77 34 34	14% (3)	74%	Na									
2003 [25]														
	22	1985-2001	No extrahepatic											
No carcinosis														
R0	8%	12/10	10/12	3/19	16	6								
1/5	0	73 38 38	20% (5)	68%	Na									
2003 [41]														
	36	1979-2001	Na	Na	16/20	17/19	10/16	Na	Na	0	64, 43, 26	11% (4)	83.3%	Na
2005 [29]														
	11	1988-1996	No extrahepatic											
No carcinosis														
Solitary nodules														
R0	Na	8/3	Na	2/9	11	0	0	73, 42, 27	18% (2)	80	Na			
2007 [34]														
	42	1985-2005	No extrahepatic											
No carcinosis														
R0	17%	20/22	Na	7/35	29	13	0	76, 48, 42	20% (8)	67%	1-86			
2007 [44]														
	37	1990-2005	No extrahepatic											
R0														
No carcinosis	12%	16/21	10/27	5/32	21	16								
9/7	14%	60 27 11	6% (2)	81%	Na									
2008 [24]														
	24	1988-2002	No carcinosis											
R0	Na	15/9	Na	8/16	13	11								
5/6	25%	38, 16, 10	8% (2)	65%	1-67									
2008 [40]														
	18	1989-2004	No extrahepatic											
R0														
Hepatic function	Na	11/7	8/10	4/14	14	14	Na	56.3, 36.5, 27	17% (3)	Na	2-200			
2008 [37]														
	22	1995-2005	No extrahepatic											
No carcinosis														
R0														
Hepatic function	7.5%	18/4	7/15	3/19	18	4								
3/1	Na	77 30.4 23	15% (3)	63.6%	1-106									
2009 [47]														
	17	1991-2005	No extrahepatic											
No carcinosis														
≤ 5 lesions														
R0	Na	9/8	Na	3/14	Na	Na	0	30.8	25% (4)	70.5%	1-117			
2009 [50]														
	73													
(11)*	1990-2004	R0												
No extrahepatic														
metachronous	15.1%	0/11	Na	1/10	8	3	0	81, 30, 20	18.2% (2)	63%	4-86			
2009 [47]	72													
(12)*	1991-2005		16.6%	12/0	Na	4/8	9	3	1	57, 43, 43	20% (3)	Na	Na	
Choi 2010														
	14	1986-2007	No extrahepatic											
No carcinosis														
R0	Na	0/14	Na	4/10	9	5								
2/3	0	67 38.3	8% (1)	63%	Na									
Tsujmoto 2010														
	17	1980-2007	No extrahepatic											
No carcinosis														
Unilobar														
R0	Na	9/8	Na	6/11	13	4	Na	31	30% (5)	70%	Na			
Our data														
2012	21	1998-2007	No extrahepatic											
No carcinosis														
R0	31%	12/9	10/11	4/17	12	9								
4/5	10%	68 31 19	14.2% (3)	66%	6-90									

H1, Metastases limited to one lobe; H2, few scattered metastases in both liver lobes; H3, numerous scattered metastases in both lobes; M, Metachronous; mets, metastases; Na, not available; S, Synchronous; STG, Subtotal gastrectomy; TG, Total gastrectomy.

● Number of patients resected on a total of patients with LMGC.

Thus, the clinical benefit of resection of hepatic metastases from gastric carcinoma is still not widely accepted. However, non-surgical treatments, including systemic or hepatic artery infusion chemotherapy, do not achieve satisfactory results. In patients treated by gastrectomy and chemotherapy, median survival times are reported to range from 2.9 to 11.8 months [37,38].

Furthermore Bines *et al*. [39] reported one long-term survivor of seven (14.3%) and other series showed 11.1% to 19% long-term survivors. Although few, the long-term survivors after hepatic resection do exist. Therefore to determine the indication of liver surgery is crucial and to clarify the condition of 5-year survivors.

The actual selection criteria, accepted by many authors (Table2) are: (1) synchronous metastases without peritoneal dissemination or other distant metastases; (2) metachronous metastases without other recurrent lesion; and (3) complete resection of metastases with acceptable postoperative liver function. Contraindications to hepatic resection are: previous extrahepatic disease, advanced lymphnode involvement, and the inability to obtain liver R0 resection (8,23). Although Roh *et al.*[28] limited the indication to one lobar distribution of metastases in a recent study, it seems that the improved patient’s survival is not observed with this limitation.

An attempt to define criteria for selection of patients with favorable outcome has been previously made in various series. We herein report a comprehensive review of the literature experience of smaller and selected population series. We classified the characteristics predictive of good or poor outcome according to the primary tumor, the metastases and the type of surgery. Table3 resumes the association of survival with different prognostic factors.

**Table 3:** Analysis of prognostic factors associated with survival in patients resected for LMGC

**Author year**	***n***	**Period**	**T**	**N**	**G**	**H**	**DIAM metastases**	**TIMING**	**MARGIN**	**MST**	**Long-term survivors**	**Recurrence**	**Follow-up (months)**
1994 [30]	21		+	+	-	-	NA	NA	NA	18	19% (4)	NA	
1997 [43]	21		-	-	-	+	NA	-	NA	NA	24% (5)	76.1%	NA
2001 [23]	17	1990-1997	-	+	+	-	NA	+	+	16	0	76%	Na
2001 [9]	40	1975-1999	-	-	-	-	-	+	-	12	15% (6)	75%	88
2001 [45]	10	1979-1999	-	-	-	-	+	+	NA	16	10% (1)	80%	10-240
2002 [33]	15	1980-1999	-	-	-	+	-	+	-	8.8	0	90%	na
2002 [31]	10	1981-1998	-	-	-	-	-	-	NA	25	20% (2)	80%	1-68
2002 [28]	19	1986-1999	-	-	+	+	-	+	NA	21	14% (3)	74%	Na
2003 [25]	22	1985-2001	*	-	-	+	+	-	NA	24	20% (5)	68%	Na
2003 [41]	36	1979-2001	-	Ly	-	+	-	-	-	NA	11% (4)	83.3%	NA
2005 [29]	11	1988-1996	NA	-	-	NA	-	-	-	19	18% (2)	80	Na
2007 [34]	42	1985-2005	+	-	-	+	-	-	-	34	20% (8)	67%	1-86
2007 [44]	37	1990-2005	+	-	-	+	+	-	-	31	6 % (2)	81 %	Na
2008 [24]	24	1988-2002	-	-	-	-	-	-	+	19	8% (2)	65%	1-67
2008 [40]	18	1989-2004	+	-	-	-	-	-	-	NA	17% (3)	Na	2-200
2008 [37]	22	1995-2005									15% (3)	63.6%	1-106
2009 [47]	17	1991-2005	-	-	-	-	-	-	+	18	25% (4)	70.5%	1-117
Choi 2010	14	1986-2007	-	-	-	-	-	-	-	NA	8% (1)	63%	Na
Tsujmoto 2010	17	1980-2007	+	Ly	NA	-	-	-	NA	34	30% (5)	70%	Na
Our data													
2012	21	1998-2007									14.2% (3)	66%	6-90

H1, Metastases limited to one lobe; H2, few scattered metastases in both liver lobes; H3, numerous scattered metastases in both lobes; M, Metachronous; mets, metastases; Na, Not available; S, Synchronous; STG, Subtotal gastrectomy; TG, Total gastrectomy; ● Number of patients resected on a total of patients with LMGC.

### Predictive of outcome related to primary tumor

Regarding the primary gastric cancer Ochiai [29] reported that hepatic resection should only be attempted in patients with synchronous or metachronous metastases if there is non-serosal invasion by the primary gastric tumor, as well as the study of Morise [40] and if the primary tumor has neither microscopic venous not lymphatic invasion in case of metachronous cases. Furthermore Shirabe showed that lymphatic and venous invasion of cancer cells from primary gastric cancer are clinicopathological prognostic factors of poor outcome at both univariate and multivariate analysis [41]. Imamura [12] add to these parameters, the grade of differentiation of primary tumor as a negative predictor of outcome. Zacherl *et al.*[32] reported that tumor localization of primary gastric cancer (proximal third versus distal two-thirds of the stomach) was a marginal predictive negative factor for overall survival of patients, while in the study of Tsujimoto the gastric cancer size > of 6 cm was considered a predictor of poor survival [42].

However, some studies showed these were not significant prognostic factors and are still controversial. Miyazaki [43] and Okano [27] reported that there was a non-significant difference in terms of depth of invasion or lymph node metastases of the gastric cancer between surviving and non-surviving patients. Koga [33] reported a marginal significance of the serosal invasion of the primary tumor.

### Predictive of outcome related to metastases

#### Number of nodules and lobar distribution

The number of the metastatic nodules in the liver has been reported to be an important prognostic factor in six studies. Okano *et al*. [27] reported 3-year survival rates of 56% for single metastases and 0% for multiple metastases, and the number of liver metastases was a significant prognostic factor in other reports as well (Table1). In Koga *et al*. and Shirabe *et al*. [33,40] none of the patients with multiple gastric liver metastases (three or more lesions) survived beyond 3 years, whereas the 5-year survival rate for patients with solitary liver metastases was 55% with eight long-term survivors. Shirabe *et al*. [40] described the presence of three or more tumors as an independent poor prognostic factor according to both univariate and multivariate analysis; moreover, all four patients who survived beyond 5 years in their study also had solitary tumors, and almost all patients described as long-term survivors (Table1) had a solitary liver metastasis. These data were confirmed in the study of Sakamoto [24] with a survival of 56% for solitary lesions against no long-term survivors in cases of multiple tumors. In a more recent study Sakamoto [44] showed against the value of solitary lesion adding unilobar distribution as a good predictive factor for survival of patients, as previously reported in the Miyazaki’s paper [42].

In some studies the number of liver metastases was a marginal prognostic factor for survival after hepatic surgery with curative intent. The favorable survival outcome for patients with a solitary metastasis, which was no worse than that for a solitary metastasis of colorectal cancer, indicates that patients with a solitary metastasis of gastric cancer are good candidates for surgical resection . On the other hand, the surgical indications should be considered more carefully in patients with multiple metastases of gastric cancer than patients with multiple metastases of colorectal cancer. Otherwise Saiura [30] showed two long-term survivors with more than three metastases concluding that if curative resection (R0) can be achieved, hepatic resection should not be abandoned even in patients with multiple liver metastases.

As for the lobar distribution of liver metastases, patients with bilobar tumors had a worse outcome than patients with a unilobar tumor, as shown by Zacherl *et al*. [32]. However, the number and lobar distribution of the tumors were correlated, and so the significance of the lobar distribution of tumors as a prognostic factor should be revaluated in larger series.

#### Histologic characteristics of liver metastases

Lymphocytes aggregation, enclosing the metastatic tumor, is reported as a good prognostic factor by Fujii [45]. This could be explained with the favorable action of umor infiltrating lymphocytes (TILs) in preventing tumor extension in gastric cancer patients [46]. Okano [27] demonstrate that the presence of a fibrous pseudocapsule around liver metastases is a promising indicator of a better prognosis, being closely associated with patient survival. Pseudocapsule formation should be considered as a protective immunoinflammatory reaction against the metastastic nodule reflecting the host defence reaction creating a wall which stops tumor diffusion.

### Predictive of outcome related to surgery

Surgical margin >/10 mm in hepatic resection was a good prognostic factor in some papers (Table2). Miyazaki [42] demonstrated significant differences in the number of hepatic metastases (solitary of multiple) and the size of the tumor-free resection margin (<10 mm or >10 mm) for long- and short-term survivors. Thelen [24] reported that a positive resection margin should be considered a powerful determinant of poor outcome. Nomura [47] showed that the recurrence rate in the remnant liver was higher in patients with a surgical margin <5 mm.

The consensus seems to be that there is not apparent value to surgery if residual disease remains, whether it is involvement of resection margins, other distant metastases, or peritoneal carcinosis.

The relationship between the extent of hepatic resection and prognosis has not yet been established. Isono [48] reported that micrometastases around the macroscopic tumor were found more frequently in hepatic metastases from gastric cancer than in those from colorectal ones, thus suggesting that wider surgical resection margins are required.

A positive resection margin is also not an independent prognostic factor in colorectal liver metastases because of its strong relationship with the number of tumors resected. In approximately 70% of patients, recurrent disease developed after hepatic resection, most commonly in the liver. Recurrent tumors were more frequently distributed in both lobes than in the resected lobe, suggesting that liver recurrence is more probably derived from multiple metastatic foci from the primary disease than from intrahepatic remetastases of the liver lesion

The paper by Nomura [46] underlines the role of intrahepatic micrometastases around the liver as a cause of recurrence of the disease. The survival rate of patients with micrometastases was significantly worse than that of patients without. In addition, recurrence after hepatic resection is more strongly associated with systemic spread through vessel infiltration than with local spread through lymphatic or serosal invasion of the primary tumor. A generous surgical margin may not be essential for curative hepatic resection of liver metastases, even if in the study by Ambiru [9] a margin <10 mm is considered a poor prognostic factor for survival. Nevertheless a positive surgical margin should be avoided and the surgeon should strive to obtain an adequate margin, because this is the only prognostic factor on which the surgeon could have any influence over. According to the pattern of recurrence, relapse developed most commonly in the liver (70%, range 63.6% to 83.3%), indicating that the remaining liver should be a focus for relapse monitoring. Early or high recurrence rates translate into prolonged periods of systemic therapy and potentially reduced quality of life: these will mitigate any short-term advantage resulting from liver resection in LMGC. Data about recurrence made liver resection less appealing. However, the majority of patients analyzed did not receive adjuvant chemotherapy after liver resection. Given the biology and systemic nature of LMGC, this may partially account for the high recurrence rates. A sensible strategy for improving survival could be close observation for a second relapse in the liver and adjuvant chemotherapies after surgery. Trials using the FOLFOXIRI regimen (5-fluorouracil, leucovorin, oxaliplatin, and irinotecan) [20] achieved a median overall survival of 15 months. We can assume that the recurrence rates could potentially decrease with these regimen in an adjuvant setting.

#### Timing of hepatic resection

Timing of hepatic resection has been reported to be a significant prognostic factor. In some paper synchronous hepatectomy was a significant poor prognostic factor. Ambiru [9] reported significantly longer survival in patients with metachronous metastases than in those with synchrounous disease (29% *vs.* 6% at 3 years). Bines [38] commented that synchronous resection of hepatic metastases has little value, whereas metachronous resection of isolated lesions can produce meaningful long-term survival, when the procedure renders the patient disease-free. Some authors suggest that resective treatment may be indicated only for the patient with metachronous isolated metastases [29,42]. Other studies did not demonstrate any differences in terms of survival among the groups (Cheon) [36]. Nevertheless, an analysis of the data reported in the recent literature showed that 22 of 48 5-year survivors underwent a synchronous hepatectomy. In fact in Sakamoto’s study [43] three of five patients who survived more than 3 years had synchronous solitary metastases and Ochiai too reported three 5-year survivors with synchronous disease. Recently Tsujimoto [41] confirm the absence of statistically difference between metachronous or synchronous nodules. Thus, synchronous hepatectomy should not be a contraindication for hepatic resection. With regard to perioperative morbidity, Bines [38], observed that synchronous liver resection carries a higher risk. This may depend on the concern regarding the use of aggressive liver surgery in conjunction with the treatment of gastric cancer under synchronous conditions. The disease-free interval (DFI) between gastric and hepatic resection has been reported to be a prognostic factor. Fujii [44] showed that a DFI > 1 year has a significant survival advantage, due to the slow growing nature of these tumors.

However, data concerning long-term survivors demonstrate that, if we exclude bilobar spread of metastases (H3), none of the previous cited predictive factors (alone or in combination) can deprive a patient of the possibility of long-term survival after hepatic resection, raising concern about the clinical value of prognostic factors emerging from small and selected populations submitted to liver resection.

The correct approach can be extrapolated from papers that addressed the topic analyzing unselected populations of gastric cancer patients presenting hepatic metastases as only site of metastatic disease (Table4). From a cohort of 58 patients Cheon [36] did not identify any primary-related or metastasis-related factor showing prognostic value. The same conclusion is done by group of Makino [49] from Japan, who studied 63 patients. Ueda [50] studied a cohort of 73 patients presenting synchronous metastases. Their data show that factors influencing survival were the extent of hepatic involvement (H1-2 *vs.* H3) and macroscopic peritoneal dissemination (P0 *vs.* P1) detected at surgical exploration. When focusing on the subgroup of H1-2 and P0 patients, they showed that number (1 *vs.* >1) and size of hepatic metastases and N status of gastric cancer (N0-1 *vs.* N2-3) were predictors of survival. An Italian survey performed under the auspices of the Italian Research Group on Gastric Cancer [51] studied an unselected cohort of 73 patients presenting metachronous metastases after curative gastrectomy. This study demonstrated that the factors T, N, and G of the gastric primary, when rated T3b-T4, N +, and G3, independently display a clear negative prognostic value with an effect that is cumulative. All the above mentioned studies strongly suggest that the main factor influencing long-term survival (*P* ranging from 0.01 to 0.001) is the therapeutic approach to the liver metastases, in particular when a surgical approach is performed. In the Italian study hepatectomy was associated to a five-fold increase in survival of less favorable patients (>1 negative prognostic factor) and achieved a 5-year survival rate of 20%. Comparing the treatment options (supportive care, chemotherapy, and liver resection) they found that 1-, 3-, and 5-year survival rates were as follows: 22%, 2%, and 0% for supportive care, 45%, 6%, and 0% after chemotherapy, and 81%, 20%, and 20% after liver resection, with a median survival as follow: non-treated patients 5 months, 12 if chemotherapy was employed and 23 months after surgery. Thus the therapeutic approach to liver metastases displayed independent significant association with survival. In the study of Ueda [49] in which a group of patients with synchronous LMGC underwent surgery or other treatments (chemotherapy, supportive cures), 1-, 3-, and 5-year overall survival rates were 57%,43%, and 43%, 60%, 0%, and 0%, and 16.7%, 0%, and 0%, respectively. Furthermore in this study adding hepatic artery infusion (HAI) chemotherapy to liver surgery not seem to offer a survival benefit for the patients. The author suggests that only liver surgery, but not HAI, could significantly prolong the survival period in this cohort of patients. Moreover the degree of liver metastases (H1-H2 *vs.* H3) seems to be a very strong prognostic factor, with a median overall survival of 16.6, 10.2, and 4.4 months, respectively,despite the treatment.

**Table 4:** Prognostic factors from series considering unselected populations and related survivals

**Author year**	**Number**	**Timing**	**MST (months)**	**1-, 3-, 5-year survival rates**	**Prognostic factors**
2008 [37]	58	Synchronous + metachronous	Overall: 16	No hepatic resection: 29.4%; 0%; 0%	
Hepatic resection RFA: 75.3%; 31.7%; 20.8%	Ro resection				
2009 [50]	72	Synchronous	NA	No hepatic resection: 36.4%; 0%; 0%	
Hepatic resection (HAIC): 80%; 60%; 60%	H; P; R0 resection				
2009 [51]	73	Metachronous	Overall: 7		
BST: 5					
Chemotherapy: 12					
Hepatic resection: 23	BST: 22%; 2%; 0%				
Chemotherapy: 45%; 6%; 0%					
Hepatic resection; 81%; 20%; 20%	T; N; G of primary				
R0 resection					
2009 [52]	73	Synchronous			Stage of primary; H
Extrahepatic disease; treatment of mets					
2010 [49]		Synchronous + metachronous	Overall: 16		
Hepatic resection: 31.2	No hepatic resection: 53.2%; 4.2%; 0%				
Hepatic resection: 82.3%; 46.4%; 37.1%					

Furthermore, two studies [36,49] underlined that R0 *vs.* R1 operation affects long-term survival (overall 5-year survival rates of 60% and 20%, respectively). Multimodal treatments can further enhance survival rates, in particular if modern chemotherapy protocols are employed. An interesting 75% 5-year survival rate in a subgroup of eight patients submitted to radical surgery followed by hepatic artery infusion chemotherapy has been reported by Ueda. Radio-frequency ablation is another important strategy for the treatment of hepatic metastases from gastric cancer. This ablative technique can be employed either as alternative or in association to hepatectomy. It could be the approach of choice in case of poor general conditions contraindicating surgery. The number of reported procedures is low not allowing to draw any conclusion on its efficacy: moreover follow-up are short and data cannot always be effectively extrapolated from the series. However, Hwang [52], considered 72 patients with metachronous metastases submitted to different treatments other than hepatectomy (Table4). The paper showed that 15 patients without extrahepatic disease treated by RFA ± chemotherapy displayed a median survival of 22 months, with 3- and 5- year survival rates of 50% and 40%, respectively, similar to those reported in surgical series (Table2). Yamakado [53] and Cheon [36] reported similar data: in their experience a subgroup of nine patients submitted to RFA compared favorably with 22 patients submitted to radical surgery, with a 4-year survival of 40% and 20%, respectively. Another paper of Kim *et al.*[54] report worse survival results similar to those of classic systemic chemotherapy alone.

## Conclusions

Some hold the view that metastatic gastric cancer represent a systemic disease and the ‘tip of the iceberg’ of a diffuse cancer, and surgery has no role in its treatment, because the results of liver resection are still disappointing. Otherwise 11% of patients survived more than 5 years after hepatectomy, are tumor-free more than 5 years after liver resection, and the identification of favorable indicators of outcome could improve these results. The key to success is to clearly identify the patients which could benefit of this treatment, in order to offer a chance to cure the patients who have good prognostic factors and to avoid overtreatment in case of absence of these factors. Moreover analysis of long-term survival reported in literature shows that, if we exclude cases presenting a bilobar spread of metastases, none of the reported predictive factors, alone or in combination, can deprive a patient of the possibility of long-term survival after hepatic resection. We believe that surgery could provide a benefit and should be a part of multidisciplinary approach in patients with liver metastases from gastric cancer.

Compared to supportive treatment alone with a median survival of 3 to 5 months, the survival figures reported in literature indicate that liver resection can improve the prognosis of patients suffering from metastatic gastric cancer, and results seems to be better than those achieved with chemotherapy alone. Data regarding the use of radiofrequency are interesting even if yet under development. This is true not only in Eastern experience, but also in Western countries, and in centers with skills and experience in liver surgery
